# Aberrant expression of Bcl2L12 as a potential biomarker for predicting recurrence in nasal polyp

**DOI:** 10.1016/j.bjorl.2025.101670

**Published:** 2025-07-04

**Authors:** Ping Zhang, Fengjun Wang

**Affiliations:** a Hunan Provincial People’s Hospital (The First Hospital Affiliated With Hunan Normal University), Department of Ultrasound, Changsha, China; b Xiangya Hospital of Central South University and Hunan Province Key Laboratory of Otolaryngology Critical Diseases, Department of Otolaryngology Head and Neck Surgery, China

**Keywords:** Chronic rhinosinusitis with nasal polyps, Recurrence, Bcl2L12, Biomarker

## Abstract

**Objective:**

Bcl2L12 plays an important role in the inflammatory response, but its role in chronic rhinitis with nasal polyps (CRSwNP) is unknown. We aimed to evaluate the relationship of Bcl2L12 with eosinophilic inflammation in CRSwNP and its value in predicting postoperative recurrence.

**Methods:**

We included 80 patients with CRSwNP treated with Functional Endoscopic Sinus Surgery (FESS), and 40 Health Control (HC) subjects. The expression levels of Bcl2L12 in tissues were examined by RT-PCR, Western Blotting (WB) and Immunohistochemistry (IHC). Correlation analysis was performed to explore the relationship between the expression level of Bcl2L12 and eosinophilic inflammation of the disease. Receiver Operating Characteristics (ROC) curves were used to assess the relationship between Bcl2L12 levels and postoperative recurrence.

**Results:**

RT-PCR results suggested that tissue Bcl2L12 levels were higher in the relapse CRSwNP group than in the control and primary CRSwNP group. Correlation analysis showed that elevated Bcl2L12 levels were positively correlated with disease eosinophilic inflammation (p < 0.05). In addition, tissue Bcl2L12 levels were higher by WB and IHC in the CRSwNP group than in the HC group (p < 0.05), especially in the recurrent group, and were centrally expressed in the epithelial region of the mucosa. Further regression analysis showed that blood eosinophil count and percentage, tissue eosinophil count, and tissue Bcl2L12 levels were closely associated with disease recurrence after surgery. The ROC curve suggested that tissue Bcl2L12 had a higher value in predicting postoperative recurrence.

**Conclusion:**

Our study shows that Bcl2L12 is increased in patients with CRSwNP, and is associated with eosinophilic inflammation. Tissue Bcl2L12 levels may be an objective marker to predict the risk of postoperative recurrence.

**Level of evidence:**

Level 3.

## Highlights

• CRSwNP with eosinophilic infiltration has a high rate of recurrence.
• Tissue Bcl2L12 levels were increased in the CRSwNP group.
• Tissue Bcl2L12 levels might predict recurrence of CRSwNP.

## Introduction

Chronic Rhinosinusitis (CRS) is a chronic heterogeneous inflammatory disease of sinonasal mucosa, affecting about 8% of the population in China and 10%–15% of the population European.^1^, ^2^ CRS can be further divided into two phenotypes: CRS with nasal polyps (CRSwNP) and CRS without nasal polyps(CRSsNP).^3^, ^4^ Compared with CRSsNP, CRSwNP is mainly characterized by T-helper-2 (Th2) inflammation with eosinophilic infiltration, which leads to more severe clinical symptoms, poorer therapeutic response, and easy recurrence after surgery.^5^, ^6^, ^7^ Previous studies have found that the recurrence rates of CRSwNP at 6-months, 24-months, and 12-years after surgery are approximately 35%, 55%, and 80%, respectively, which places a heavy burden on both society and patients.^8^, ^9^, ^10^ In view of this, accurate preoperative differentiation of patients susceptible to recurrent CRSwNP is essential for selecting appropriate treatments and improving follow-up programs. Stewart and colleagues found that the severity assessed on preoperative Computed Tomography (CT) scans was an important predictor of treatment outcome,^11^ but this way was lack objectivity. Therefore, it is crucial to explore objective biomarkers to predict postoperative recurrence of CRSwNP, which can help clinicians develop effective treatment strategies and improve patient prognosis.

B-cell Lymphoma protein-2 Like-12 protein (Bcl2L12) is a member of the Bcl-2 family, which is commonly involved in the regulation of apoptosis and cell survival.^12^ Studies suggest that Bcl2L12 may regulate immune cell activity involved in the development of inflammatory diseases.^13^, ^14^ Li et al.^15^ found that Bcl2L12 contributes to Th2-biased inflammation in the intestinal mucosa by regulating CD4 + T-Cell. Furthermore, Yang and his colleagues showed that Bcl2L12 facilitates experimental airway allergic inflammation by inducing autocrine eotaxin in eosinophils.^16^ However, the role of Bcl2L12 in CRSwNP is currently unknown. We hypothesized that Bcl2L12 might be associated with eosinophilic inflammation in CRSwNP. To test this hypothesis, nasal polyp tissue were collected from patients with CRSwNP. The expression of Bcl2L12 in tissues was analyzed; the relationship between Bcl2L12 expression and eosinophilic inflammation was explored and whether it could be used as a biomarker for predicting recurrence after CRSwNP.

## Methods

### Population and study design

We included 80 patients with CRSwNP, including 40 primary CRSwNP (pCRSwNP) and 40 recurrent CRSwNP (rCRSwNP) patients. They underwent Functional Endoscopic Sinus Surgery (FESS) between December 2023 and April 2024 at our medical center. In addition, we recruited 40 healthy patients as a control group. We followed the inclusion criteria outlined in the European Position Paper on Rhinitis and Nasal Polyps.^1^ CRSwNP exclusion criteria were as follows: patients with fungal sinusitis, sinus tumors, systemic inflammatory diseases, autoimmune disorders, or eosinophilic disorders; and patients who had taken immune-modulating medications, antibiotics, oral or topical corticosteroids, or antiallergic medications in the 4-weeks prior to the procedure. We performed routine preoperative examinations and collected demographic and clinical data. We assessed patients' subjective symptoms using a Visual Analog Scale (VAS). In addition, we assessed preoperative computed tomography scores according to the Lund–Mackay scale and preoperative endoscopy scores according to the Lund-Kennedy scale. This prospective study was approved by the ethical committee of our hospital. All participants signed informed consent.

### Assessment of postoperative recurrence

In this study, nasal polyp recurrence included pre-inclusion recurrence (some of whom had undergone surgery) and pre-inclusion recurrence-free but with symptoms of recurrence at follow-up. Patients with no recurrence before inclusion all received a uniform postoperative care plan that included nasal saline rinses, oral antibiotics, and topical corticosteroids. CRSwNP recurrence was defined as the reappearance of clinical symptoms with evidence of endoscopy persisting for at least 2-months despite salvage therapy with antibiotics and oral steroids.

### Real-Time Polymerase Chain Reaction (RT-PCR) analysis

Tissue samples from patients were collected during surgery. Total RNA was isolated from nasal polyp tissues using TRIzol (Invitrogen, USA). Then 1 μg of total RNA was used as the initial material, and cDNA was synthesized using a reverse transcription kit (Qiagen, Hilden, Germany), and reverse transcription polymerase chain reaction (RT-PCR) was performed using SYBR Premix EX Taq (UE, Suzhou, China). The expression of target genes was standardized by 3-Phosphoglycerate Dehydrogenase (GAPDH), and the data were analyzed by the 2^−ΔΔCT^ method. The primer sequences as fellow: GAPDH, Forward primer: CATGGCACCGTCAAGGCTGAGA, Reverse primer: TCCTAGTTGCCTCCCCAAAGCACA. Bcl2L12, Forward primer: TGTTTGAGGAGCAGGGAGGAGGA, Reverse primer: GGCGCCCATTAGCGCCTAAATC.

### Western Blotting (WB)

Western blotting was performed as previously described.^17^ Total tissue proteins were extracted in RIPA lysis buffer containing protease inhibitors. Equal amounts of proteins were subjected to SDS-PAGE electrophoresis in 10% Tris-glycine gels and then transferred to PVDF membranes (Millipore, Massachusetts, USA). The membranes were closed with 5% skimmed milk for 1 h at room temperature and then incubated overnight with Bcl2L12 and microtubule protein primary antibody (Affinity Biosciences, Changzhou, China). The membrane was then incubated with HRP-conjugated secondary antibody (1:3000; Bioworld, Nanjing, China) for 1 h. The bands were developed using ECL reagent (Advansta, California, USA). The relative intensity of each band was normalized to the relative intensity of GAPDH.

### Immunohistochemistry (IHC)

Nasal tissue samples were fixed with 4% paraformaldehyde to prepare paraffin sections, and then manipulate as previously described.^18^ Sections were blocked with 10% goat serum for 30 min at room temperature. Then, they were incubated overnight at 4 °C with antibodies against Bcl2L12 (Affinity, Changzhou, China) at a dilution ratio of 1:500. The samples were incubated overnight at 4 °C and then incubated with HRP-labeled secondary antibody (1:2000; Affinity, Changzhou, China) for 1 h at room temperature. Sections were visualized under a microscope (Leica, Wetzlar, Germany). To quantify the data, the integrated optical density (IOD) of positive tissue expression was measured using ImageJ software (National Institutes of Health, Bethesda, MD, USA) and compared using IOD/area values.

### Statistical analysis

Statistical analyses were performed using SPSS Statistics version 25.0 and GraphPad Prism version 8.3.0. Data were expressed as mean ± SD if they obeyed normal distribution; otherwise, they were expressed as median (25‒75th percentile). Comparisons between the two groups were performed using Student's *t*-test or Kruskal-Wallis H-test. Pearson's test or Spearman's test was used to determine correlation. Cox logistic regression analysis was performed to determine independent predictors of postoperative recurrence. The researcher plotted the Receiver Operating Characteristic Curve (ROC) and calculated the Area Under the Curve (AUC). p-value <0.05 was considered statistically significant.

## Results

### Comparison of clinical characteristics of patients

As shown in Table 1, blood eosinophil counts and percentages were higher in the CRSwNP group than in the control group. While other clinical variables were not significantly different between the two groups. Table 2 further demonstrates that compared to pCRSwNP, blood and tissue eosinophil counts, Lund-Kennedy and Lund–Mackay scores were elevated in the rCRSwNP group. No significant differences in other clinical variables were observed between the two groups.

**Table 1:** Demographic characteristics between HC group and CRSwNP group.

Clinical variables	HC group (n = 40)	CRSwNP group (n = 80)	p-value
Male/Female	25/15	49/31	0.894
Age, years	39.3 ± 14.7	42.7 ± 15.1	0.242
BMI, Kg/m^2^	21.6 ± 3.8	22.5 ± 4.1	0.215
Smoking, yes/no	9/31	21/59	0.655
Drinking, yes/no	6/34	20/60	0.210
Peripheral blood EOS count, 10^6^/L	233.3 ± 147.9	427.5 ± 135.0	<0.001
Peripheral blood EOS percentage (%)	4.1 ± 2.9	8.0 ± 2.2	<0.001

HC, Health Control; CRSwNP, Chronic Rhinosinusitis with Nasal Polyp, BMI, Body Mass Index; EOS, Eosinophil.

**Table 2:** Demographic characteristics between pCRSwNP and rCRSwNP patients.

Clinical variables	pCRSwNP group (n = 40)	rCRSwNP group (n = 40)	p-value
Male/Female	23/17	26/14	0.491
Age, years	42.4 ± 14.4	43.0 ± 16.0	0.849
BMI, Kg/m^2^	22.8 ± 4.1	22.2 ± 4.0	0.521
Smoking, yes/no	8/32	13/27	0.204
Drinking, yes/no	9/31	11/29	0.606
Peripheral blood EOS count, 10^6^/L	365.0 ± 151.1	490.0 ± 77.8	<0.001
Peripheral blood EOS percentage (%)	7.3 ± 2.3	8.6 ± 2.0	0.008
Tissue EOS count, n/HPF	12.5 ± 9.2	23.8 ± 20.5	0.002
Tissue EOS percentage (%)	10.6 ± 7.8	13.4 ± 9.3	0.148
VAS score	6.3 ± 2.0	7.0 ± 2.0	0.103
Lund-Kennedy score	8.0 ± 2.6	11.1 ± 5.4	0.002
Lund-Mackay score	10.4 ± 4.4	12.9 ± 6.6	0.047

pCRSwNP, Primary Chronic Rhinosinusitis with Nasal Polyp; rCRSwNP, Recurrent Chronic Rhinosinusitis with Nasal Polyp; BMI, Body Mass Index; EOS, Eosinophil; VAS, Visual Analogue Scale.

### Exploring the expression level of Bcl2L12 in and its correlation with eosinophilic inflammation

**Fig. 1:**
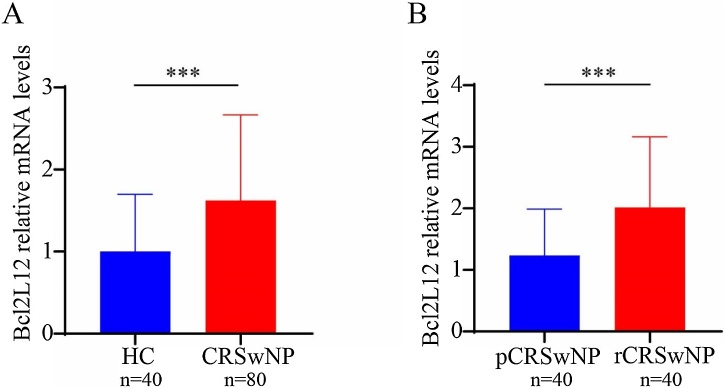
Tissue expression level of Bcl2L12 in the CRSwNP patients. (A) Tissue Bcl2L12 expression levels were significantly higher in the CRSwNP group than in the HC group. (B) Compared to the pCRSwNP group, the expression level of Bcl2L12 were obviously elevated in the rCRSwNP group. HC, Healthy Controls; CRSwNP, Chronic Rhinosinusitis with Nasal Polyp. ***p < 0.001.

**Fig. 2:**
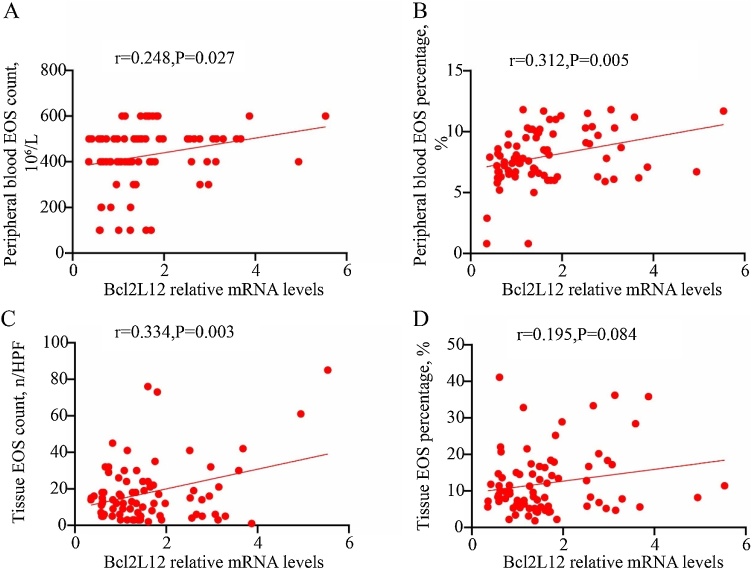
Correlation between Bcl2L2 expression levels and eosinophilic inflammation in CRSwNP patients. (A‒C) Tissue Bcl2L2 mRNA expression levels were significantly and positively correlated with w peripheral blood EOS counts and percentages as well as tissue EOS counts; (D) While there was no significant correlation with tissue EOS percentages. CRSwNP, Chronic Rhinosinusitis with Nasal Polyp; EOS, Eosinophil.

**Fig. 3:**
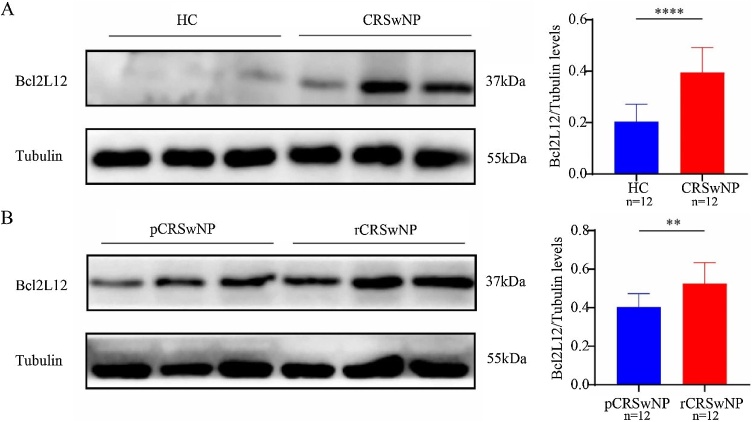
The protein level of Bcl2L12 in the CRSwNP patients. (A) WB images of Bcl2L12 levels were significantly increased in the CRSwNP group than in the HC group. (B) Bcl2L12 levels were significantly enhanced in the rCRSwNP group than in the pCRSwNP group. HC, Healthy Controls; CRSwNP, Chronic Rhinosinusitis with Nasal Polyp. **p < 0.01, ****p < 0.0001.

**Fig. 4:**
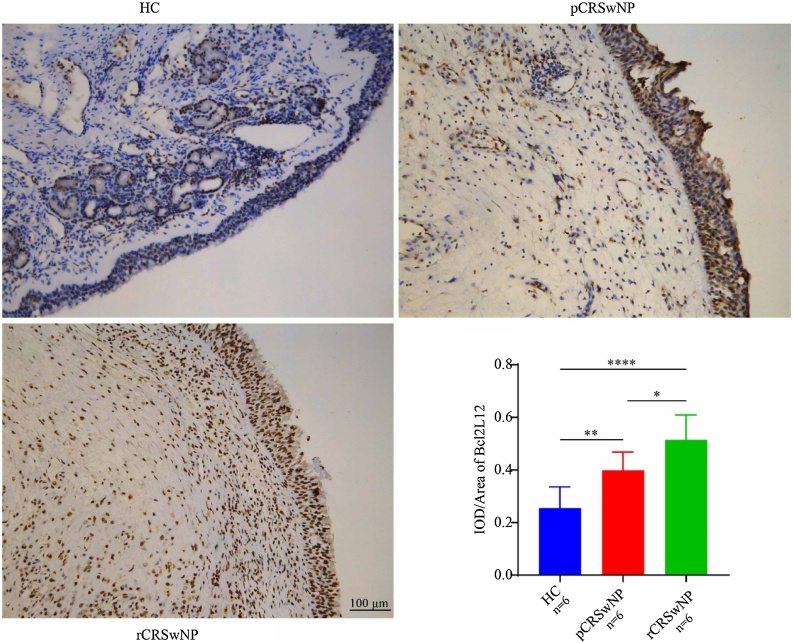
Localization of tissues Bcl2L12 expression in CRSwNP patients. HC, Healthy Controls; CRSwNP, Chronic Rhinosinusitis with Nasal Polyp. *p < 0.05, **p < 0.01, ****p < 0.0001.

As shown in Fig. 1, RT-PCR results showed that tissue Bcl2L12 mRNA levels were significantly higher in the CRSwNP group compared with the HC group. Further analysis revealed that the level of Bcl2L12 was clearly increased in the rCRSwNP group with respect to the pCRSwNP group. In addition, Correlation analysis in Fig. 2 revealed that the mRNA levels of Bcl2L12 in tissues of CRSwNP patients were significantly and positively correlated with serum eosinophil counts, percentages, and tissue eosinophil counts. Moreover, the WB results further revealed that Bcl2L12 protein expression levels in tissues were remarkably increased in the CRSwNP group, especially in recurrent CRSwNP (Fig. 3). The IHC staining demonstrated that Bcl2L12 were mainly expressed in nasal epithelial cells and submucosal (Fig. 4).

### Evaluation of the predictive value of tissue Bcl2L12 for CRSwNP recurrence

**Fig. 5:**
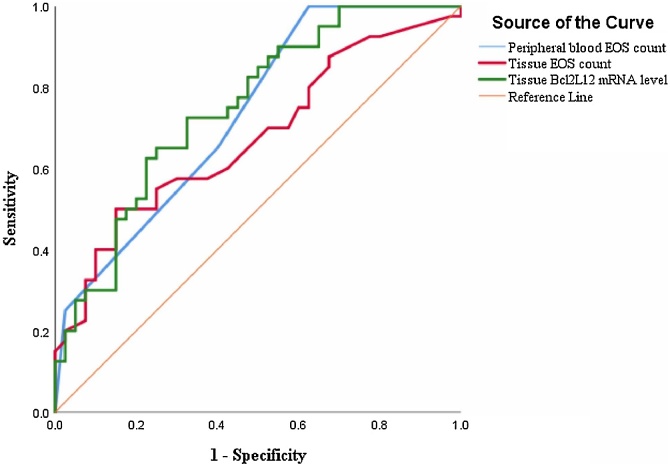
ROC curves for predicting CRSwNP recurrence. ROC, Receiver Operating Characteristic; CRSwNP, Chronic Rhinosinusitis with Nasal Polyp.

To further explore the clinical factors associated with disease recurrence, we included the index of variance from Table 2 and Bcl2L12 mRNA levels in a binary regression model, as shown in Table 3, which showed that peripheral blood and tissue eosinophil counts, as well as tissue Bcl2L12mRNA levels were risk factors for CRSwNP recurrence. Subsequently, we further found by ROC analysis in Fig. 5 that tissue Bcl2L12 mRNA levels had higher predictive value for postoperative recurrence of CRSwNP compared to blood and tissue eosinophil count. Detailed data in Table 4.

**Table 3:** Logistic regression analysis of factors associated with CRSwNP recurrence.

Clinical variables	OR	95% CI	p-value
Peripheral blood EOS count, 10^6^/L	1.009	1.003‒1.015	0.003
Peripheral blood EOS percentage (%)	1.254	0.948‒1.658	0.112
Tissue EOS count, n/HPF	1.053	1.007‒1.102	0.025
Tissue Bcl2L12 mRNA levels	2.589	1.410‒4.753	0.002

CRSwNP, Chronic Rhinosinusitis with Nasal Polyp; EOS, Eosinophil; OR, Odds Ratio; CI, Confidence Interval.

**Table 4:** ROC Analysis results of indicators for predicting the recurrence of CRSwNP.

Clinical variables	AUC (95% CI)	Cut-off value	Sensitivity	Specificity	p-value
Peripheral blood EOS count, 10^6^/L	0.733 (0.624‒0.841)	350.0	90.0%	37.5%	<0.001
Tissue EOS count, n/HPF	0.675 (0.557‒0.793)	18.5	50.0%	85.0%	0.007
Tissue Bcl2L12 mRNA levels	0.751 (0.646‒0.857)	1.3	72.5%	67.5%	<0.001

CRSwNP, Chronic Rhinosinusitis with Nasal Polyps; EOS, Eosinophil; AUC, Area Under the Curve; CI, Confidence Interval.

## Discussion

Previous studies have shown that most CRSwNP patients were at high risk of postoperative recurrence,^19^, ^20^ so it is clinically important to assess the prognosis of CRSwNP patients and predict postoperative recurrence. In the present study we found that tissue Bcl2L12 levels were significantly increased in the CRSwNP group, especially in the rCRSwNP group. In addition, we found that tissue Bcl2L12 levels correlated with the degree of mucosal eosinophilic infiltration and was strongly associated with postoperative recurrence. Given that, we believed that tissue Bcl2L12 expression was involved in the development of CRSwNP and it could be used as a novel biomarker to predict postoperative recurrence.

It has been discovered that the expression of Bcl2L12 was correlated with the activity of immune cells such as eosinophil cells, mast cells and T-cells.^12^, ^16^ Consequently, its abnormal expression is linked to immune dysregulation, which is implicated in various inflammatory diseases. Yang et al.^16^ have demonstrated that Bcl2L12 plays a crucial role in the development of allergic airway diseases by prolonging the lifespan of eosinophils and enhancing their production. Therefore, inhibiting Bcl2L12 in eosinophils holds promise as a translational approach for treating allergic airway diseases. Moreover, Li and Feng et al.^15^, ^21^ have delineated the involvement of Bcl2L12 in the pathogenesis of Inflammatory Bowel Disease (IBD) by regulating CD4 + T-cell function, consequently driving Th2-mediated inflammation within the intestinal mucosa. These investigations collectively underscore the pivotal contribution of Bcl2L12 to both eosinophilic inflammation and Th2-driven immune responses. Notably, it is well known that Th2-mediated inflammation and eosinophilic infiltration represent hallmark pathological characteristics of CRSwNP,^22^, ^23^, ^24^ thereby the precise interplay between Bcl2L12 expression and the pathophysiology of CRSwNP remains an area warranting further investigation. In our investigation, we observed the notable upregulation of tissue Bcl2L12 levels within the CRSwNP cohort in contrast to the HC group. Furthermore, we identified a positive correlation between tissue Bcl2L12 expression and peripheral blood and tissue eosinophil ratios. These findings suggest a potential association between heightened the abnormal expression level of tissue Bcl2L12 and the pathogenesis of CRSwNP, likely intertwined with tissue eosinophilic inflammation. However, elucidating the precise underlying mechanism warrants further investigation.

Our investigation revealed a marked elevation in tissue Bcl2L12 levels among patients with rCRSwNP compared to the pCRSwNP patients. Additionally, The ROC curves showed that tissue Bcl2L12 exhibited superior predictive capability for postoperative CRSwNP recurrence. Previous study found that Bcl2L12 was a novel biomarker for the prediction of short-term relapse in nasopharyngeal carcinoma.^25^ In addition, elevated levels of Bcl2L12 could expedite the production and secretion of chemokines by eosinophils, thereby facilitating their migratory infiltration.^26^, ^27^, ^28^ Importantly, extensive literature underscores eosinophilic infiltration and Th2 inflammation as established risk factors for CRSwNP recurrence following surgical intervention.^29^, ^30^, ^31^ Notably, eosinophilic infiltration into tissue emerges as a pivotal contributor to CRSwNP recurrence. Moreover, elevated Bcl2L12 exacerbates type 2 inflammation by promoting the differentiation of CD4 + T cells into the Th2 phenotype.^32^, ^33^ Consequently, we posited that environmental stimuli activate and enhance Bcl2L12 secretion by nasal mucosal epithelial and inflammatory cells within nasal polyps. Elevated Bcl2L12 levels likely exacerbate eosinophilic infiltration into nasal mucosal tissues by augmenting Th2 cell differentiation and cytokine production, thereby intensifying type 2 inflammation and eosinophilia within nasal mucosal tissues and heightening the risk of CRSwNP recurrence. These findings collectively underscore the involvement of tissue Bcl2L12 in the eosinophilic inflammatory response in CRSwNP and highlight its clinical significance in predicting postoperative recurrence.

In summary, significantly elevated tissue Bcl2L12 levels in CRSwNP patients were associated with postoperative recurrence. Tissue Bcl2L12 could be used as a novel objective biomarker to predict postoperative recurrence in CRSwNP. However, the results of this study have not been confirmed by further large-sample, multicenter studies. In addition, the involvement of Bcl2L12 in the recurrence mechanism after CRSwNP needs to be further explored.

## ORCID ID

Fengjun Wang: 0009-0004-7904-9647

Ping Zhang: 0009-0001-4156-4270

## Ethics approval and consent to participate

This study was conducted in accordance with the recommendations of Declaration of Helsinki. The Human Ethical Committee of Xiangya Hospital of Central South University approved this study (2023111316), and all participants provided informed consent.

## Funding

This study was supported by the Natural Science Foundation of Hunan Province (2020JJ4910).

## Data availability

The datasets used and/or analyzed during the current study are available from the corresponding author on reasonable request.

## Declaration of competing interest

The authors declare no conflicts of interest.
